# The Endoscopic Treatment of Carpal Tunnel Syndrome as an Outpatient Procedure

**DOI:** 10.1155/DTE.4.183

**Published:** 1998

**Authors:** T. Buchhorn, E. A. Cameron, H. G. Klausmann, C. Erggelet, J. Krämer

**Affiliations:** 1 Orthopaedic Surgery Dr. Klausmann Macairestrasse 19 Konstanz D-78467 Germany; 2 Praxisklinik Rennbahn St. Jakobsstrasse 106 Muttenz CH-4132 Switzerland; 3 Department of Orthopaedics Universityclinic of Freiburg Hugstetterstrasse 55 Freiburg D-79106 Germany; 4 Department of Orthopaedics Universityclinic of Bochum Gudrunstr. 56 Bochum D-44791 Germany; 5 Kleelistr.1 Scherzingen CH-8596 Switzerland

## Abstract

From September 1995 to July 1996 50 patients were treated for carpal tunnel syndrome as outpatients by endoscopic release in the rooms of an orthopaedic surgeon (two-portal-technique). The average age was 51.3 years (27–61 years). The average length of symptoms was 43 months, the postoperative time off work averaged 27 days. Six months postoperatively wasting of the thenar persisted in 2 out of 16 patients, a positive Tinel's sign in 1 out of 46 patients and delayed median nerve conduction in 2 out of 48 presenting these symptoms preoperatively. At 6 months the average handgrip strength had recovered to 109% of the preoperative value. One out of 49 patients still presented paresthesia and 1 out of 50 nocturnal dysesthesia. There were minor complications in 7 patients (14%), only one patient requires further treatment. We conclude that endoscopic carpal tunnel release done on outpatients in a private surgery can be reliable, safe and cost efficient.


*Diagnostic and Therapeutic Endoscopy*, Vol. 4, pp. 183–190
Reprints available directly from the publisher
Photocopying permitted by license only

© 1998 OPA (Overseas Publishers Association)
Amsterdam B.V. Published under license
under the Harwood Academic Publishers imprint,
part of The Gordon and Breach Publishing Group.
Printed in Singapore.

### The Endoscopic Treatment of Carpal Tunnel Syndrome as an Outpatient Procedure

T. BUCHHORN[a,*], E.A. CAMERON[b], H.G. KLAUSMANN[a], C. ERGGELET[c] and J. KRÄMER[d]

[a] *Orthopaedic Surgery Dr. Klausmann, Macairestrasse 19, D-78467 Konstanz, Germany;* [b] *Praxisklinik Rennbahn, St. Jakobsstrasse 106, CH-4132 Muttenz, Switzerland;* [c] *Department of Orthopaedics, Universityclinic of Freiburg, Hugstetterstrasse 55, D-79106 Freiburg, Germany;* [d] *Department of Orthopaedics, Universityclinic of Bochum, Gudrunstr. 56, D-44791 Bochum, Germany*

*(Received 26 June 1997; In final form 10 November 1997)*

**From September 1995 to July 1996 50 patients were treated for carpal tunnel syndrome as outpatients by endoscopic release in the rooms of an orthopaedic surgeon (two-portal-technique). The average age was 51.3 years (27–61 years). The average length of symptoms was 43 months, the postoperative time off work averaged 27 days. Six months postoperatively wasting of the thenar persisted in 2 out of 16 patients, a positive Tinel's sign in 1 out of 46 patients and delayed median nerve conduction in 2 out of 48 presenting these symptoms preoperatively. At 6 months the average handgrip strength had recovered to 109% of the preoperative value. One out of 49 patients still presented paresthesia and 1 out of 50 nocturnal dysesthesia. There were minor complications in 7 patients (14%), only one patient requires further treatment. We conclude that endoscopic carpal tunnel release done on outpatients in a private surgery can be reliable, safe and cost efficient.**

*Keywords:* Carpal tunnel syndrome, Endoscopy, Surgery, Outpatient, Complications, Chow technique

### INTRODUCTION

The carpal tunnel syndrome (CTS) is the most common nerve compression syndrome [1] accounting for approximately 20% of all peripheral nerve lesions. In 50% of all patients it is the cause of brachialgia. The prevalence amongst women as quoted from [2] is 9.2% and amongst men 0.6%. It is therefore commonly seen in private practice. Table I summarises disorders predisposing to CTS.

The carpal tunnel syndrome can be classified into 3 stages. Stage 1 is defined as mainly nocturnal burning or tingling pains of the hand and/or forearm (dysesthesia nocturna). In stage 2 there is thenar wasting and hypesthesia and hypalgesia

* Corresponding author. Kleelistr.1, CH-8596 Scherzingen, Switzerland. Tel.: ++41 71 6887066. Fax: ++41 71 6887066.

TABLE I Predisposing conditions of carpal tunnel syndrome

| | |
|---|---|
| 1. Trauma | Fractures |
| | Carpal bone dislocations |
| | Recurrent microtrauma |
| 2. Endocrine dysbalances | Pregnancy |
| | Climacterium |
| | Hypothyroidism |
| | Acromegaly |
| 3. Systemic illnesses | Gout |
| | Diabetes |
| | Amyloidosis |
| | Lupus erythematodes |
| | Haemophilia |
| | Rheumatoid arthritis |
| 4. Others | Tumours |
| | Tenontitis |
| | Malformations |

following the distribution of the sensory fibres of the median nerve. Stage 3 finally is characterised by trophic and vasomotor disturbances of the affected hand.

The patient's history and the clinical lead to the correct diagnosis findings [3,4]. The severity of the compression can be quantified by measuring the motor latency of the median nerve [5]. If the patient's history is suggestive of traumatic genesis or of neoplastic origin, standard X-rays and MRI are advocated [5,6]. The initial treatment of CTS is conservative with splinting at night, systemic NSAID's or local injections of cortisone. Herskovitz *et al.* [7] reported good short-term results after oral administration of steroids. Conservative treatment though rarely offers lasting relief and marked wasting of the thenar, once present, can persist even after decompression. So surgery should not be delayed for too long when conservative therapy is not successful [8].

Endoscopic carpal tunnel release has rapidly gained popularity over the last 8 years. Okutsu *et al.* first presented the technique in 1989. They used a translucent tube which was advanced from the forearm into the carpal tunnel to view the sectioning of the carpal ligament through an endoscope introduced into this tube.

In 1990 Chow reported his endoscopic technique. He used a two-portal-technique and a V-shaped gutter, through which endoscope and cutting devices are introduced into the carpal tunnel and the ligament split under vision.

In 1992 Agee *et al.* described a single portal-technique with the skin incision at the wrist. Cutting devices and endoscope are therefore all introduced proximally to distally. The ligament is sectioned in a retrograde fashion by a mechanically operated cutting device.

Endoscopic treatment of carpal tunnel syndrome has now become the standard operative treatment at specialised clinics [11–15]. The morbidity as quoted in the literature [16] is very low. So, if the rate of complications remained low when this technique is performed by a specialist on an outpatient basis in his surgery, it would make it a cost efficient way of treating CTS.

### MATERIALS AND METHODS

### Patients

Fifty patients who were operated on from September 1995 to July 1996 in one orthopaedic surgeon's surgery form the basis of this study. Patients with post-traumatic carpal tunnel syndrome were excluded from the study. All patients were questioned about their work and classified according to Baldasseroni *et al.* [17] into "job-classes".

### Clinical Workup

Thenar wasting was evaluated clinically. The presence of Tinel's sign was registered [4]. A balloon manometer was used to measure handgrip strength. The motor conduction latency of the median nerve was determined preoperatively, at one and four weeks and then at six months postoperatively. A conduction latency value under 4.5 m/s was considered to be normal, over 4.5 m/s or over 1.5 m/s difference to the contralateral side as pathological. The following additional clinical parameters were registered: Paresthesia, dysesthesia nocturna and a subjective rating of the intervention. Time off work was noted.

### Operation Technique

All 50 operations were done under local anesthesia. When the patient felt uncomfortable with the instruments *in situ* i.v.-analgesics were administered.

The patient is then positioned supine, the arm abducted 90° on an armboard. The landmarks are drawn on the skin [18] (Fig. 1). Then the arm is disinfected. Under local anesthesia [19] the proximal incision is made first at the site marked previously (Fig. 2). The palmar aponeurosis is split longitudinally exposing the fascia. A narrow dissector is advanced under the transcarpal ligament, preparing the path for the following instruments. By feeling for the rough undersurface of the carpal ligament the surgeon makes sure the dissector is positioned correctly under the ligament. At this stage of the operation the patient often feels some discomfort. Next the trocar follows the path of the dissector (see Fig. 3) with the wrist maximally hyperextended over the specially designed bolster (Fig. 4). The second skin incision is made over the tip of the trocar (Fig. 5). Now the transverse carpal ligament should be visible at the 12 o'clock position if the instrument has been placed correctly (Fig. 6). Special knives are used to dissect the ligament, the dissection should be continued for 1–1.5 cm into the fascia of the forearm. Once the ligament is dissected subcutaneous fat bulges into the gutter (see Fig. 7) compromising the endoscopic view. Swabs help to improve the view.

The operation is finished by probing the boarders of the ligament. The trocar is reintroduced into the gutter and both removed from the carpal tunnel. The skin is sutured with non-resorbable 6.0 material. A well padded dressing is applied and left for three days postoperatively. The

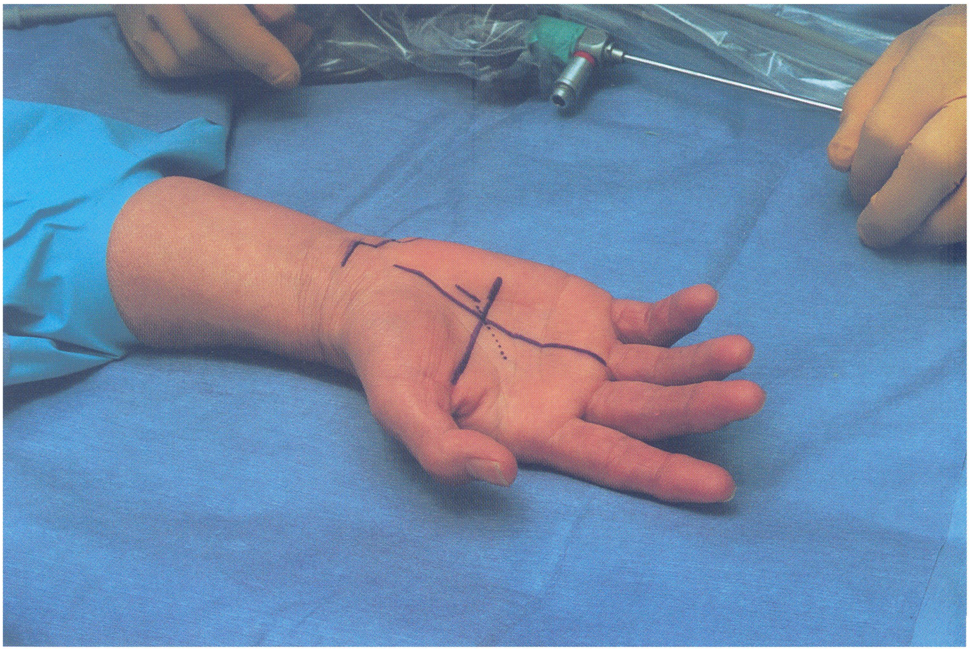

FIGURE 1 Landmarks drawn on skin preoperatively.

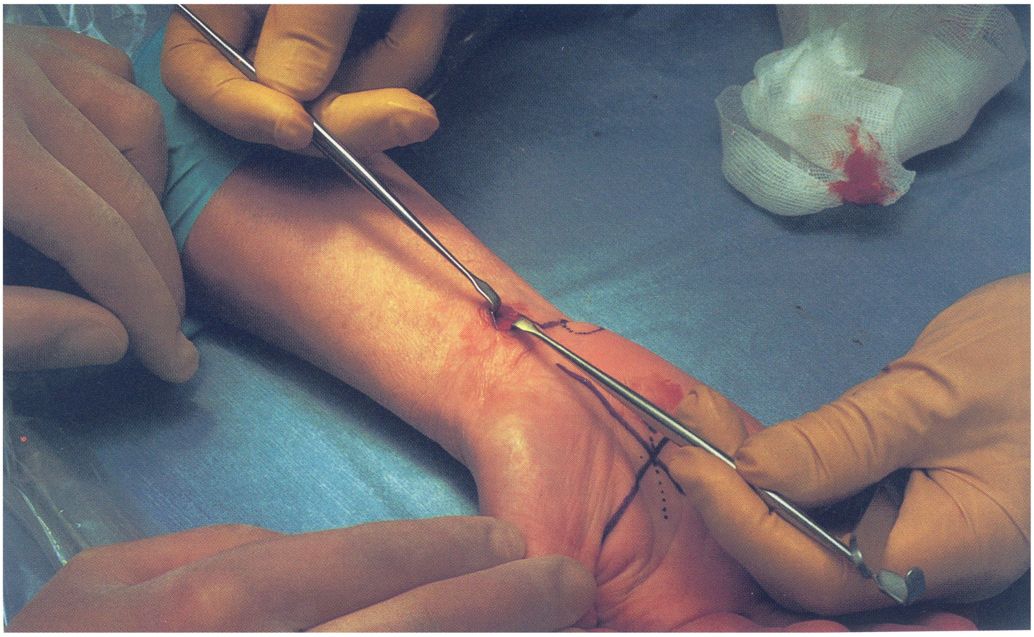

FIGURE 2 Proximal skin incision.

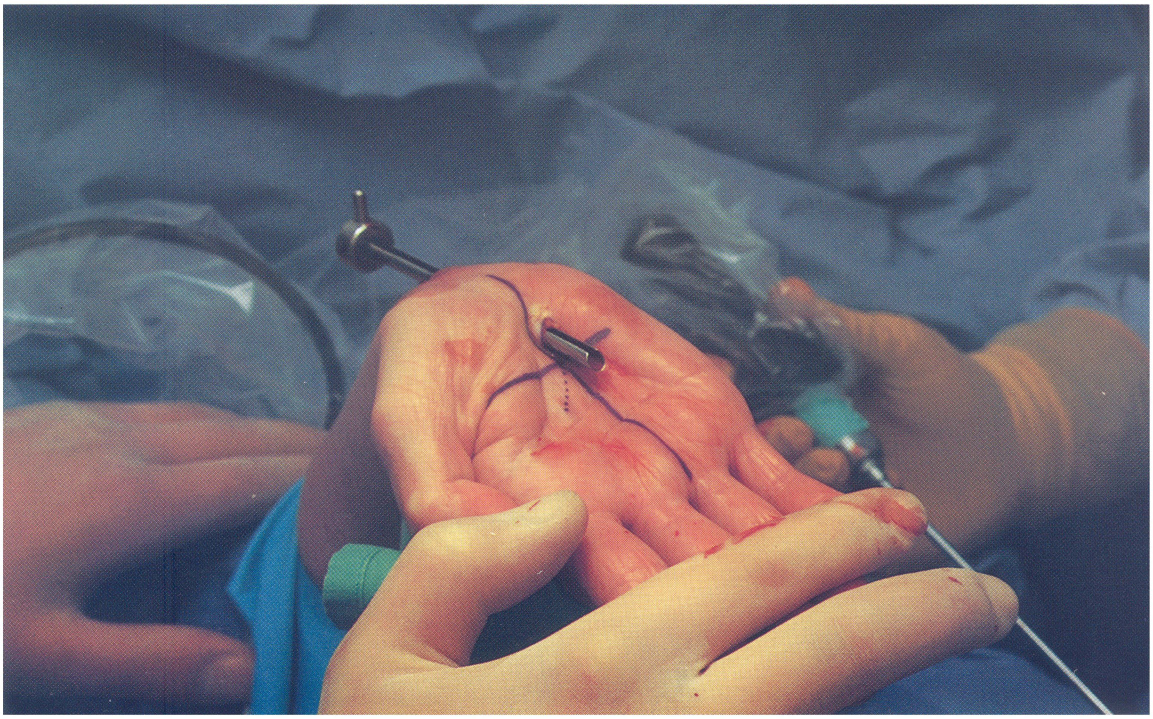

FIGURE 3 Trocar and instrument gutter *in situ*.

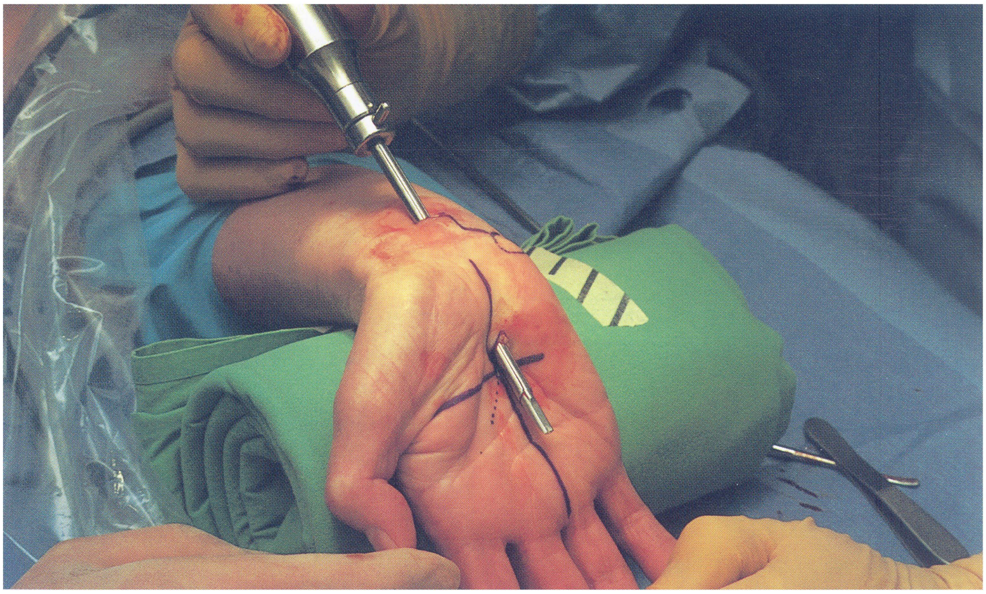

FIGURE 4 Wrist hyperextended over bolster.

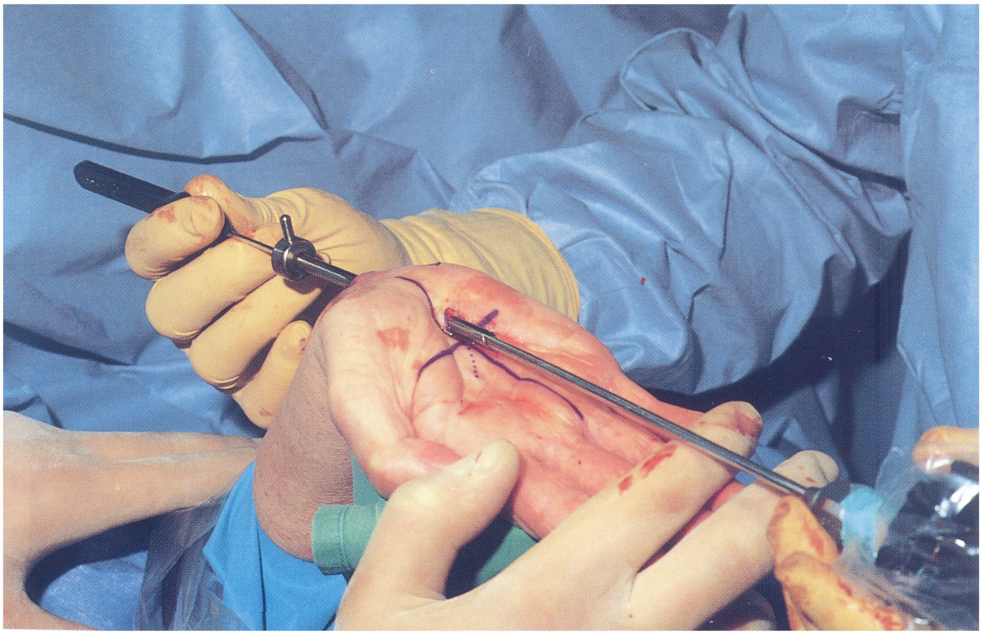

FIGURE 5 Distal skin incision.

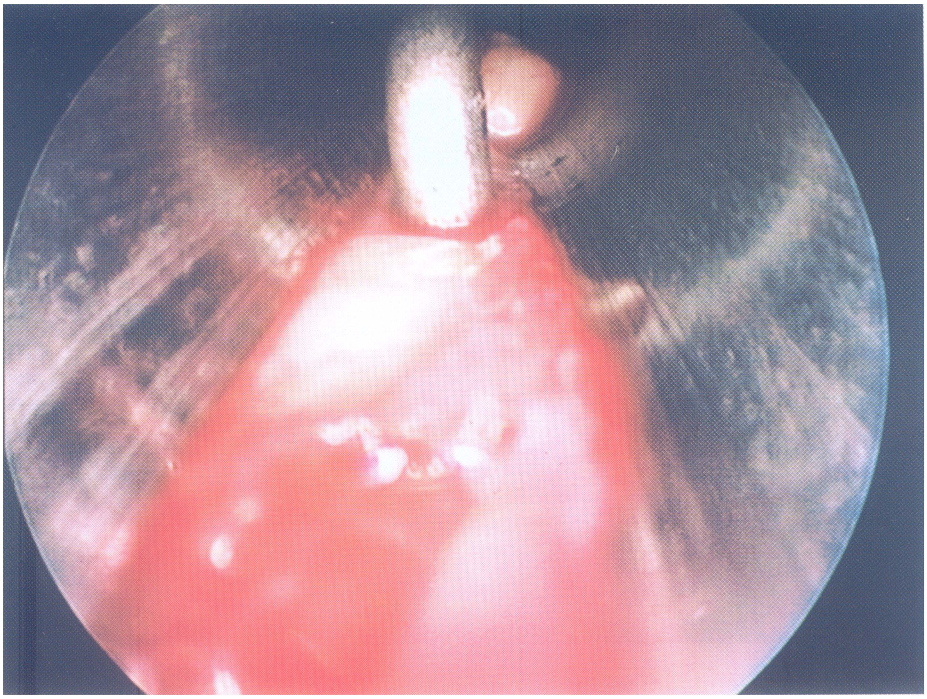

FIGURE 6 Transverse carpal ligament visible over gutter.

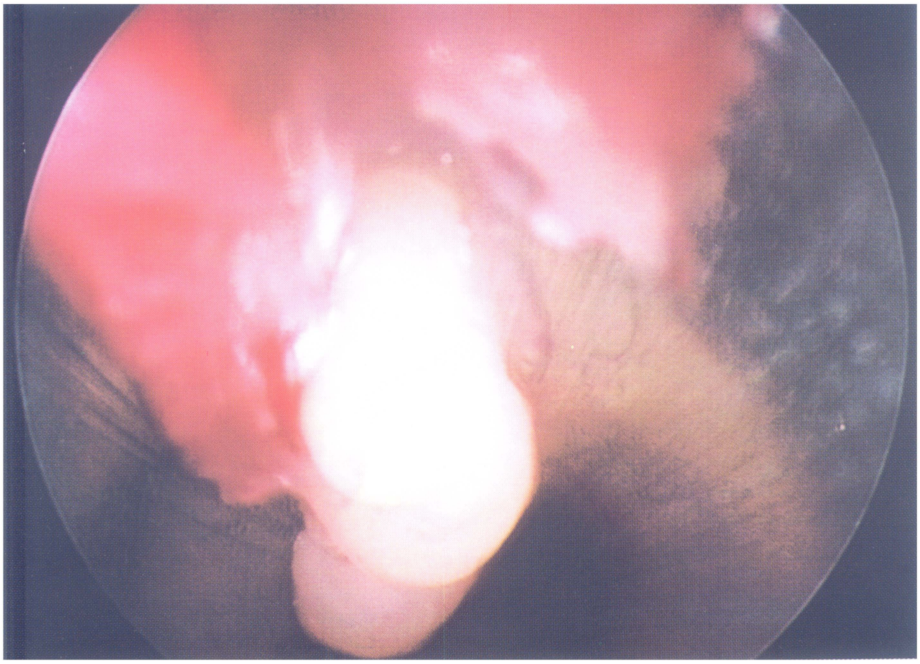

FIGURE 7 Subcutaneous fat bulging into gutter after sectioning of ligament.

patients receive no physiotherapy. On demand, the patients could take systemic NSAID's.

### RESULTS

### Demografic and Clinical Data

Twenty-nine of the 50 patients were female, 21 male. Twenty eight times the right wrist and 22 times the left wrist and twice both wrists were operated on. The average age was 51.3 years (27–61 years). Five patients could be classified as hard manual labourers, 8 as manual labourers, 21 as average labourers and 16 were light workers. The average duration of symptoms prior to surgery was 43 months. The average time off work was 27 days. Tables II–VII summarise the clinical and neurological data. All patients claimed they would opt for the same endoscopic treatment.

TABLE II Wasting or thenar

| | Preoperatively | 1 week postop. | 4 weeks postop. | 6 months postop. |
|---|---|---|---|---|
| No wasting | 39 | 39 | 39 | 48 |
| Little wasting | 7 | 7 | 9 | 2 |
| Marked wasting | 4 | 4 | 2 | — |
| Severe wasting | — | — | — | — |

TABLE III Tinel's sign

| | Preoperatively | 1 week postop. | 4 weeks postop. | 6 months postop. |
|---|---|---|---|---|
| Positive | 46 | 7 | 4 | 1 |

TABLE IV Motor latency of median nerve

| | Preoperatively | 1 week postop. | 4 weeks postop. | 6 months postop. |
|---|---|---|---|---|
| Pathologically elevated | 48 | 48 | 35 | 2 |

TABLE V Handgrip strength (in % of preoperative value)

| 1 week postop. | 4 weeks postop. | 6 months postop. |
|---|---|---|
| 52 | 84 | 109 |

TABLE VI Paresthesia

| Preoperatively | 1 week postop. | 4 weeks postop. | 6 months postop. |
|---|---|---|---|
| 49 | 16 | 3 | 1 |

TABLE VII Dysesthesia nocturna

| Preoperatively | 1 week postop. | 4 weeks postop. | 6 months postop. |
|---|---|---|---|
| 50 | 11 | 2 | 1 |

### Complications

There were no intraoperative complications. Three patients developed a hematoma postoperatively. All but 1 patient were free of symptoms at six months p.o. This 1 patient presented with persisting paresthesia and requires revision surgery. In 2 patients the motor latency of the median nerve hat not recovered to normal at six months postoperatively. As they are free of symptoms this cannot be considered to be a complication in the strict sense. Three patients reported sore scares for several months. All in all, 7 patients presented with minor complications.

Chow described 1993 no permanent nerve or vessel damage, hematoma, tendon laceration or infection found in his series. The comparison between outpatient and hospital based inpatient shows no increase in complications rate of the outpatient procedure.

### DISCUSSION

When discussing results of studies on CTS one must stress the fact that interpreting neurophysiological data is not without problems. The motor latency of the median nerve is influenced both by the investigator and by circadiem rhythm. The latency also increases with age [16] and is influenced by hormonal changes. Considering these facts, functional impairment, pain characteristics and clinical findings determine the indication for operation. Statistical analysis of the pre- and postoperative neurophysiological data therefore makes little sense. More important is the clinical outcome and the patient's satisfaction with the procedure, which was very good in this series. The only patient with persisting symptoms shows fibrosis of the epineurium of the median nerve on MRI, the aetiology of which cannot be determined.

With an average 27 days off work our series compared favourably with data reported by Agee *et al.* in 1992. In his series the average time off work was 25 days after unilateral endoscopic procedures. However, as the patients in this study did not receive any postoperative physiotherapy [20] we feel that routine physiotherapy could have shortened the time back to work. This question remains to be studied by further investigations.

Although 4 patients presented marked wasting of the thenar and 7 moderate wasting all of them demonstrated some recovery after six months. In such cases the literature [16] favours open procedures. It is noteworthy though, that in none of the patients in this series the function of the thumb was impaired. If this had been the case more extensive procedures would have been necessary to restore the opposition movement of the thumb [21,22].

Endoscopic techniques need to be thoroughly instructed before they can be practised safely [15,21,22]. Once mastered, this technique is simple and cost efficient due to short operating times and reusable and resterilisable instruments.

We therefore conclude that the endoscopic release of the carpal ligament can be practised safely and cost efficiently as an outpatient procedure in private practice. It thus offers a valuable alternative to the open procedure performed on hospital based inpatients.

### *References*

[1] Brandt, T., Dichgans, J. and Diener, H.C.: *Therapie und Verlauf neurologischer Erkrankungen*. Kohlhammer Verlag. Stuttgard-Berlin, 1988.

[2] De Krom, M.C., Knipschild, P.G., Kester, A.D. *et al.*: Carpal tunnel syndrome: prevalence in general population. *J. Clin. Epidemiol.* 1992; **45**: 373–376.

[3] De Smet, L., Steenwerckx, A., van den Bogaert, G. *et al.*: Value of clinical provocative tests in carpal tunnel syndrome. *Acta Orthop. Belg.* 1995; **61**(3): 177–182.

[4] Seror, P.: Sensitivity of the various tests for the diagnosis of carpal tunnel syndrome. *J. Hand Surg.* 1994; **19**(6): 725–728.

[5] Britz, G.W., Haynor, D.R. and Kuntz, C.: Carpal tunnel syndrome: correlation of MRI, clinical, electrodiagnostic and intraoperative findings. *Neurosurgery* 1995; **37**(6): 1097–1103.

[6] Mesgarzadeh, M., Triolo, J. and Schneck, C.D.: Carpal tunnel syndrome: MRI imaging diagnosis. *Magn. Reson. Imag. Clin. N. Am.* 1995; **3**(2): 249–264.

[7] Herskovitz, S., Berger, A.R. and Lipton, R.B.: Low-dose, short term oral prednisone in the treatment of carpal tunnel syndrome. *Neurology* 1995; **45**(10): 1923–1925.

[8] Kulik, R.G.: Carpal tunnel syndrome. *Ortho. Clin. N. Am.* 1996; **27**(2): 345–354.

[9] Okutsu, I., Niomiya, S., Takatori, Y. *et al.*: Endoscopic management of the carpal tunnel syndrome. *Arthroscopy* 1989; **5**: 11–18.

[10] Chow, J.C.Y.: Endoscopic release of the carpal tunnel ligament for carpal tunnel syndrome: 22 month clinical result. *Arthroscopy* 1990; **6**: 288–296.

[11] Agee, J.M., McCarroll, H.R., Tortosa, R.D. *et al.*: Endoscopic release of the carpal tunnel syndrome: a randomized prospective multicentre study. *J. Hand Surg. Am.* 1992; **17**: 987–995.

[12] Brock, M., Iprenburg, M. and Janz, C.: Die endoskopische Behandlung des Karpaltunnelsyndromes. *Dtsch. Ärzteblatt* 1994; **91**(42): 2848–2852.

[13] Brown, R.A., Geldermann, R.H. and Seiler, J.G. *et al.*: Carpal tunnel release. *J. Bone and Joint Surg.* 1993; **73A**: 1265–1275.

[14] Chow, J.C.Y.: The Chow technique of endoscopic release of the carpal tunnel ligament for carpal tunnel syndrome: four years of clinical results. *Arthroscopy* 1993; **9**: 301–314.

[15] Janeta, C., Baltzer, A., Strauss, M. *et al.*: Endoskopische Therapie bei Karpaltunnelsyndrom. *Arthroskopie* 1996; **9**: 2–10.

[16] Preissler, P.: Die palmar-dorsale endoskopische Karpalbandspaltung. *Arthroskopie* 1996; **9**: 11–16.

[17] Baldasseroni, A., Tartaglia, R. and Carnevale, F.: The risk of the carpal tunnel syndrome in some work activities. *Med. Lav.* 1995; **86**(4): 341–351.

[18] Cobb, T.K., Knudson, M.D. and Cooney, W.P.: The use of topographical landmarks to improve the outcome of Agee endoscopic carpal tunnel release. *Arthroscopy* 1996; **11**(2): 165–172.

[19] Dagrenat, P., Spaite, A., Restelli, S. *et al.*: Anésthesie loco-régionale pour endoscopique du canal carpien. *Ann. Fr. Anaest. Réanim.* 1995; **14**(3): 306–309.

[20] Weitbrecht, W.U., Schäfer, W. and Walter, A.: Ist Krankengymnastik nach Karpaltunnesyndromoperationen sinnvoll? *Z. Orthop. Ihre Grenzgeb.* 1995; **133**(5): 429–431.

[21] Lowry, W.E.: Treatment of severe compression of the median nerve. In: Gelbermann R.H. (ed.) Operative Nerve Repair and Reconstruction, J.B. Lippincott Company, Philadelphia, 1991.

[22] Skandalakis, J.E., Colborn, G.L., Skandalakis, P.N. *et al.*: The carpal tunnel syndrome: Part I–III. *Am. J. Surg.* 1992; **58**: 72–81, 158–166.

